# Thermoresponsive GenisteinNLC-dexamethasone-moxifloxacin multi drug delivery system in lens capsule bag to prevent complications after cataract surgery

**DOI:** 10.1038/s41598-020-80476-x

**Published:** 2021-01-08

**Authors:** Tingyu Yan, Zhongxu Ma, Jingjing Liu, Na Yin, Shizhen Lei, Xinxin Zhang, Xuedong Li, Yu Zhang, Jun Kong

**Affiliations:** 1 Department of Ophthalmology, The Fourth Affiliated Hospital of China Medical University, No.11 Xinhua Road, Heping District, Shenyang, 110005 Liaoning Province China; 2 Tianjin Eye Hospital, Tianjin Key Laboratory of Ophthalmology and Vision Science, Clinical College of Ophthalmology, Tianjin Medical University, No. 4 Gansu Rd, Heping District, Tianjin, 300020 China; 3 Department of Pharmaceutics, Shenyang Pharmaceutical University, No.103 Wen Hua Road, Shenyang, 110016 China

**Keywords:** Drug delivery, Lens diseases

## Abstract

Cataract surgery is the most common intraocular procedure. To decrease postsurgical inflammation, prevent infection and reduce the incidence of secondary cataract, we built a temperature-sensitive drug delivery system carrying dexamethasone, moxifloxacin and genistein nanostructured lipid carrier (GenNLC) modified by mPEG-PLA based on F127/F68 as hydrogel. Characterizations and release profiles of the drug delivery system were studied. In vitro functions were detected by CCK-8 test, immunofluorescence, wound-healing assay, real time-PCR and western blotting. The size of GenNLCs was 39.47 ± 0.69 nm in average with surface charges of − 4.32 ± 0.84 mV. The hydrogel gelation temperature and time were 32 °C, 20 s with a viscosity, hardness, adhesiveness and stringiness of 6.135 Pa.s, 54.0 g, 22.0 g, and 3.24 mm, respectively. Transmittance of the gel-release medium was above 90% (93.44 ± 0.33% to 100%) at range of 430 nm to 800 nm. Moxifloxacin released completely within 10 days. Fifty percent of dexamethasone released at a constant rate in the first week, and then released sustainably with a tapering down rate until day 30. Genistein released slowly but persistently with a cumulative release of 63% at day 40. The thermoresponsive hydrogel inhibited the proliferation, migration and epithelial-mesenchymal transition of SRA 01/04 cells, which were confirmed by testing CCK-8, wound-healing assay, western blot, real time-PCR (RT-PCR) and immunofluorescence. These results support this intracameral thermoresponsive in situ multi-drug delivery system with programmed release amounts and release profiles to cut down the need of eye drops for preventing inflammation or infection and to reduce posterior capsular opacification following cataract surgery.

## Introduction

Cataract is the leading cause of blindness^1^, while cataract surgery is the most common and usually successful intraocular surgical procedure^2^. Potentially serious complications of cataract surgery include intraocular inflammation, infection, or posterior capsule opacification (PCO)^3^. Each year in the United States, 150,000 of these complications occur, reported in approximately 5% of patients^4,5^. Eye drops often are routinely prescribed to reduce the risk of inflammation. In clinical practice, antibiotics eye drops often are applied for a week while topical corticosteroids often are used for a month^6,7^. However, limitations of eye drop solutions, include low bioavailability, low patient compliance and poor eye drop administration technique, which could lead to ineffective treatments. If eye drops are not instilled properly, contamination of the eye dropper may occur^8^. Moreover, there are no effective eye drops for preventing PCO commercially available at this time^9^. Nd: YAG laser capsulotomy is the only effective treatment for PCO; however, there can be post-laser complications such as inflammation and retina detachment, and it has associated costs^10,11^.

Over the past several decades, many attempts have been made to develop ocular drug delivery systems to replace post-operative eye drops and therefore increase patient compliance in the post-operative period. Intracameral inserts and implants were designed to provide sustained drug delivery and to improve bioavailability. Surodex (Oculex Pharmaceuticals, Inc., Sunnyvale, CA) is a biodegradable anterior chamber insert that provides sustained-release dexamethasone for about 10 days. Surodex only completed phase II trials in the United States. It has been approved in Singapore for the treatment of postoperative inflammation following cataract surgery^12,13^. The first long-acting intracameral product designed for anterior segment approved by FDA in 2018 is DEXYCU (Icon Bioscience Inc., Newark, CA), a bioabsorbable corticosteroids drug-delivery system with dexamethasone suspended in the acetyl triethyl citrate that can be detected in aqueous humor up to 21 days^14,15^. For ophthalmic antibiotics application, there is no sustained drug delivery system currently available commercially to our knowledge. Simona. S et al*.* reported a nanocomposite intravitreal injectable delivery system that undergo sol–gel transition upon change in temperature, which was in animal testing level, to provide long lasting release of cefuroxime for preventing endophthalmitis after cataract surgery^16^. The drug delivery platforms mentioned above were designed for carrying single type of medicine and for specific indication.

In recent years, more attempts have been made towards designing multi-drug delivery systems. A thermoresponsive injectable drug depot that released levobunolol, dexamethasone and moxifloxacin was designed for postoperative treatment following cataract surgery^17^. However, applying anti-glaucoma agents is not recommended for routine use after cataract surgeries. Moreover, it was designed for intravitreous injection, which is not a typical administrative approach for anterior segment. Therefore, development of a multi-drug delivery system containing antibiotics, steroids and anti-PCO agent with special programmed release profiles and can be injected and form in situ gel in the residual lens capsule at the end of cataract surgery may be an ideal solution. This multi-drug in situ gel could potentially abrogate the needs for eye drops administration after cataract surgery and prevent PCO formation as well. As a cavity organ, eye is an optimal target for injectable temperature sensitive hydrogel. Mixture of Pluronic F127 (F127) and Pluronic F68 (F68) are widely used to construct temperature sensitive in situ gels because the proportion can be adjusted flexibly to formulate long acting system with ideal gelation temperature. The in situ gels can be injected into the eyeball as solution at room temperature, and turn into non-flowing gel once being warmed up to body temperature^18–20^. Thermoresponsive hydrogels were studied as intravitreal injectable drug delivery platform encapsulating anti-VEGF antibodies for treating age related macular degeneration (AMD) in *vitro* and in animal models by researchers^21,22^.

In our previous studies, we constructed nanostructured lipid carriers (NLCs) carrying genistein (Gen), a soy-derived biologically active isoflavone and natural occurring tyrosine kinase inhibitor, which was shown to inhibit the proliferation, migration and myofibroblast transformation of epithelial cell in the whole process of PCO formation^23–26^. In this study, the GenNLC was modified by methoxy polyethylene glycol-block-Poly (D, L-lactide) (*mPEG-PLA*), which could improve surface properties and provide sustainable genistein release^27^. However, in our in vivo study (unpublished data, under review), the longest release duration in the anterior chamber of the GenNLC was less than 10 days. We hypothesized that cooperating NLC into temperature sensitive hydrogels can further prolong genistein release profile in the anterior chamber^28^. Moreover, the existence of NLCs embedded in hydrogels could reduce the rate of gel erosion^29^. Based on the previous work, in this study, we aimed to develop a temperature-sensitive in situ hydrogel carrying genistein nanostructured lipid carrier (GenNLC), dexamethasone (Dex) and moxifloxacin (Mox), which can be injected into the anterior chamber at the conclusion of cataract surgery, especially in the paediatric population, to reduce inflammation, prevent infection and reduce the incidence rate of posterior capsule opacification. We optimized the formulation of the multi-drug delivery system to meet the designed programmed release amounts and release profiles, and evaluated the performance of the drug delivery system in vitro.

## Results

### Preparation and characterization of GenNLCs

**Figure 1:**
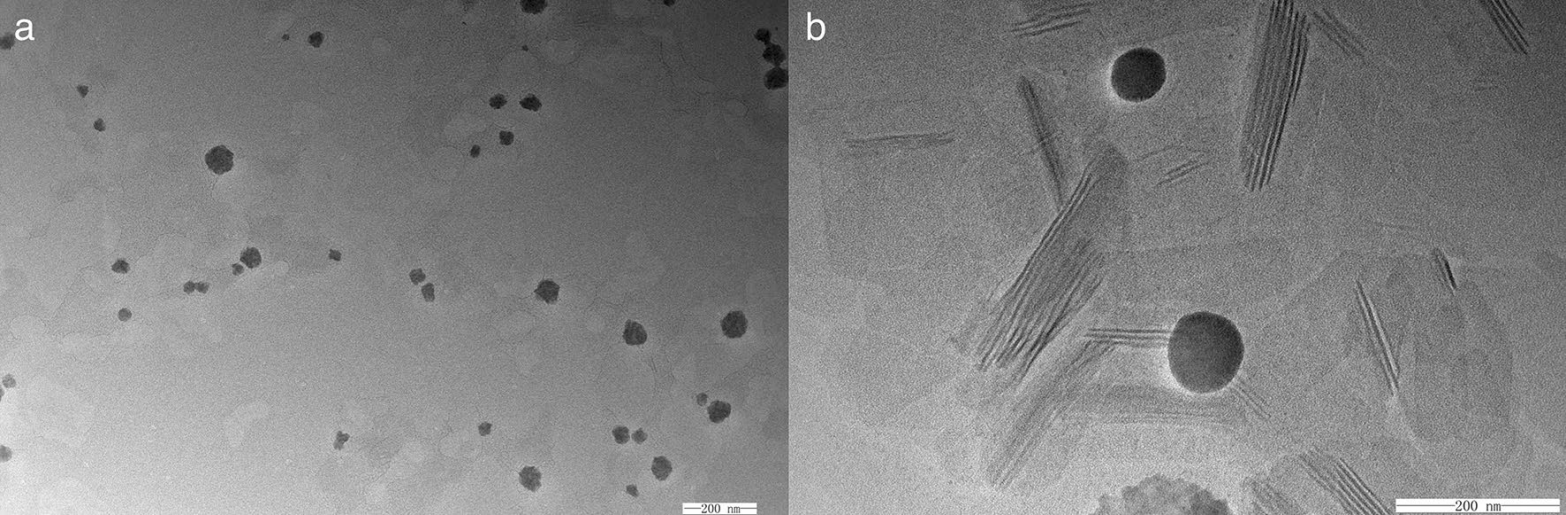
Transmission electron microscopy micrographs of the GenNLCs. (**a**) GenNLCs were monodispersed without obvious aggregation. (**b**) Particles were monodispersed without obvious aggregation, generally in spherical shapes with smooth edge, displaying solid, dense structure. Scale bar: 200 nm.

Genistein is an insoluble drug in water. High temperature emulsification and homogenization methods were used to synthase GenNLC. In order to build optimal NLCs with even high entrapment efficiency and high stability compared with the former formulations of GenNLCs built by our group previously^23–26^, we selected two solid lipids and two liquid lipids which had been well studied in our lab. The stability of GenNLCs constructed by different lipids fomulations is listed in Table 1. As shown, the duration of GenNLCs staying clear without segments ranged from less than 1 day to more than 1 month. The formulation with soybean oil and Comprital ATO 888 showed the shortest stability duration, while, the GenNLCs constructed with MCT and Compritol ATO888 stayed stabilized more than one month without aggregative sediments and significant increase of zeta potentials. MCT and Compritol ATO888 were selected as lipids for constructing the modified GenNLCs in the following experiments. The modified GenNLCs constructed by this fomulation had an ideal average size (39.47 ± 0.69 nm), moderate polydispersity index (0.213 ± 0.016), and negative Zeta potentials (− 4.32 ± 0.84 mV). The encapsulation efficiency (EE) of the GenNLCs was 92.75 ± 2.72%. As shown in the transmission electron microscope images, the morphologies of GenNLCs were monodispersed without obvious aggregation, generally in spherical shapes with smooth edge, displaying solid, dense structure (Fig. 1). The modified GenNLCs had an even smaller size, on average, than those of the GenNLCs we constructed previously (minimum: 80.12 ± 1.55 nm)^23^, without any definitive evidence of compromising the stability. This may be attributed to the modified excipients when employing mPEG-PLA, Kolliphor HS 15 and Cremphor EL as surface agents.

**Table 1:** Stability properties of the GenNLCs formulated with different combinations of liquid lipids and solid lipids (mean ± SD) (n = 3).

Lipids combination	Stability
MCT + Compritol ATO 888	> 30 days
Soybean oil + Compritol ATO 888	0.67 ± 0.29 day
MCT + Precirol ATO 5	4.83 ± 0.76 days
Soybean oil + Precirol ATO 5	7.33 ± 0.58 days

### Preparation of GenNLC-Dex-Mox thermoresponsive in situ hydrogel

**Figure 2:**
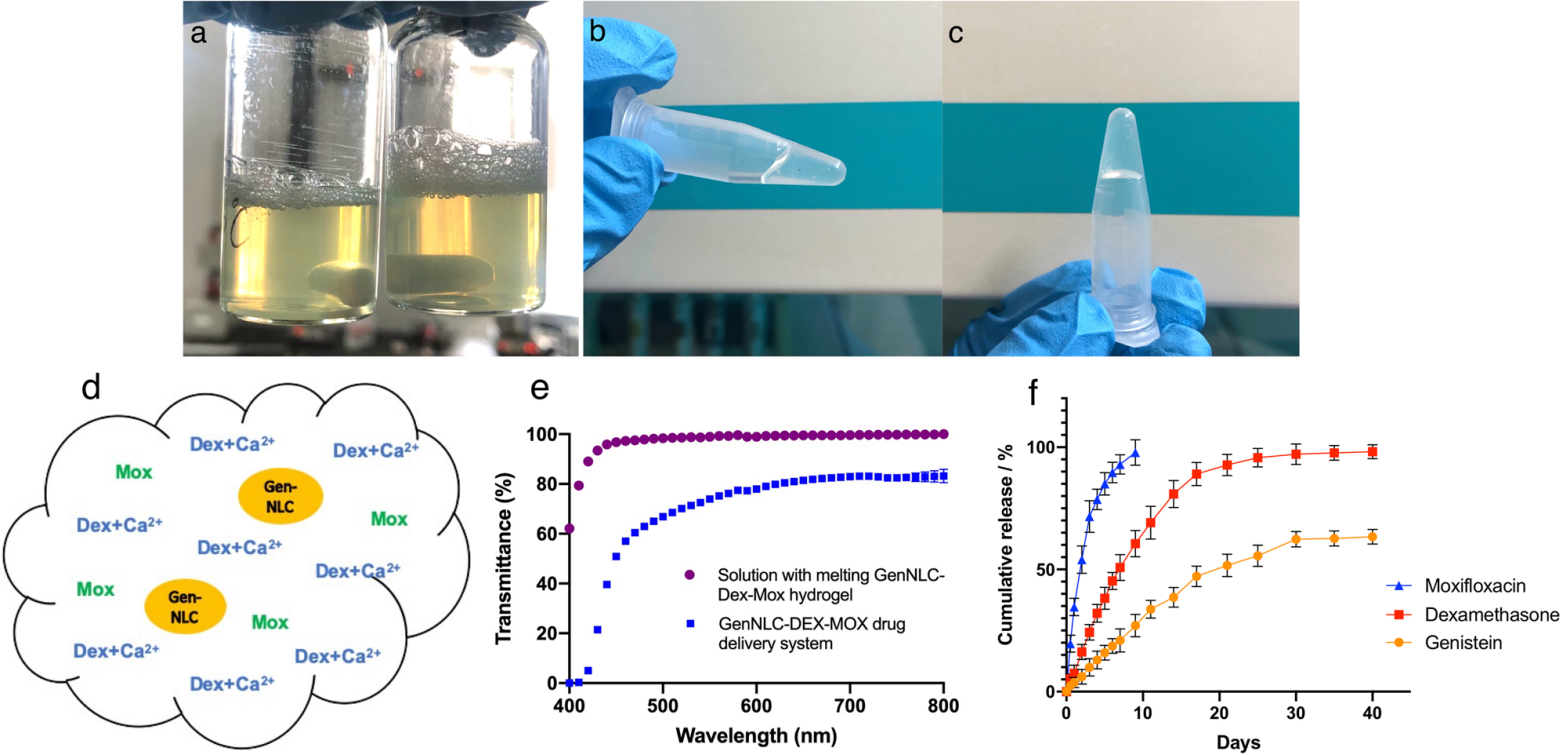
Physical appearance, sketch, transmittance and in vitro release profiles of the multi-drug delivery system containing dexamethasone (Dex), moxifloxacin (Mox) and genistein (Gen). (**a**) The hydrogel was light yellow, homogeneous and transparent in liquid phase. (**b**) The thermosensitive hydrogel presented in semi-liquid/semi-gel transition phase when it was heated up to 30 °C. (**c**) The hydrogel in gel phase when the temperature was above 33 °C. (**d**) Genistein was entrapped in nanostructured lipid carrier (GenNLC). Dex was combined with CaCl_2_. Mox was added directly into the hydrogel. (**e**) Within the range of 500–800 nm, the overall transmittance of the hydrogel (blue) was over 65% (66.88 ± 0.72%**–**83.22 ± 1.87%). The transmittance in the range of 400**–**500 nm decreased to 0.05%. The transmittance of the solution with melting GenNLC-Dex-Mox hydrogel (purple) was 62.15 ± 0.12% at 400 nm, while the transmittance were all above 90% (93.44 ± 0.33% to 100%) at range of 430 nm to 800 nm. (**f**) Moxifloxacin showed a quick release for up to 10 days and 98.76 ± 2.31% of moxifloxacin molecules were released till day 9. Dexamethasone was released from hydrogel in the first week at a high but constant rate, then, the rest released sustainably until day 30 (97.14 ± 3.38%) with a tapering down rate. Genistein was released slowly but persistently with a decreasing rate as well. The cumulative release amount of genistein was 63.35 ± 2.49% at day 40.

Different proportions of F127 and F68 were tested to formulate the thermoresponsive in situ hydrogel for the purpose of injectable and gelatinized design in the equator area of residual lens capsule bag at the end of cataract surgery. The tested concentrations of F127 ranged from 25 to 28%w/v, while the range of F68 concentrations was from 1 to 7.5%w/v. The results showed that the gelation temperatures of the gels ranged from 32.0 to 37.33 °C close to human biological temperatures with the gelation time of the gels ranged from 20 to 33 s. The ideal gelation time of injectable thermosensitive in situ gel should allow enough time to be injectable and form gel as soon as possible once it is inside the lens capsule bag. As a result, the formulation containing 26% F127 and 1.5% F68 which showed a gelation temperature of 32.0 ± 0.5 °C and a gelation time of 20.67 ± 0.58 s met the requirement best. The final formulated GenNLC-Dex-Mox temperature sensitive hydrogel was a light yellow, homogeneous and transparent (Fig. 2a) solution. It presented in liquid form at room temperature, while, it formed a non-flowing gel once being warmed up to body temperature (Fig. 2b,c). Therefore, the formulation containing 26% F127 and 1.5% F68 was selected for the further tests (Table. 2). The final concentrations of the three agents in our GenNLC-Dex-Mox temperature sensitive in situ hydrogel were 4 mg/mL of dexamethasone, 2 mg/mL of moxifloxacin, and 1 mg/mL of genistein respectively (Fig. 2d).

**Table 2:** Gelation temperature and gelation time of the different fomulations by adjusting concentrations of F127 and F68 in GenNLC-Dex-Mox delivery system (mean ± SD) (n = 3).

	F127 (% W/V)	F68 (% W/V)	Gelation temperature (°C)	Gelation Time (s)
Gel 1	28	7.5	37.33 ± 0.29	32.33 ± 2.52
Gel 2	28	7	36.5 ± 0.5	29.67 ± 1.53
Gel 3	26	2	33.0 ± 0.5	23.67 ± 1.53
Gel 4	26	1.5	32.0 ± 0.5	20.67 ± 0.58
Gel 5	25	2	33.5 ± 0.5	24.33 ± 1.53
Gel 6	25	1	35.0 ± 1.0	26.33 ± 0.58

### Characterization of GenNLC-Dex-Mox thermoresponsive in situ hydrogel

The viscosity of selected GenNLC-Dex-Mox multi-drugs delivery systems with and without CaCl_2_ were measured by quantitative rheological measurements. Based on the results, the viscosity of GenNLC-Dex-Mox gel with CaCl_2_ was 6.135 Pa.s at 25 °C, which is appropriate for administration at room temperature and moderate for ocular injection. The viscosity of the gel without CaCl_2_ was 4.336 Pa.s at 25 °C. The lower the viscosity is, the easier the gel solution is injectable in a syringe. The results of texture analyzer showed the adhesive abilities of the gels with CaCl_2_ or without CaCl_2_. The hardness, adhesiveness and stringiness data are shown in Table 3. Both of the GenNLC-Dex-Mox thermoresponsive in situ gels with or without CaCl_2_ illustrated ideal adhesive ability, while, the gel with CaCl_2_ (hardness 54.0 g, adhesiveness 22.0 g, stringiness 3.14 mm) exerted even better adhesiveness than the gel without CaCl_2_ (hardness 48.5 g, adhesiveness 20.1 g, stringiness 2.26 mm). The transmittance of GenNLC-Dex-Mox hydrogel at visible light wavelength region (400–800 nm) is shown in Fig. 2e. Within the range of 500–800 nm, the overall transmittance was above 65% (66.88 ± 0.72%–83.22 ± 1.87%). The transmittance in the range of 400–500 nm decreased to 0.05%. However, the gel was not designed to be applied in the passway of visual axis. It was designed to be injected into the equatorial part of the residual lens capsule bag, which is blocked by the pupil. Thus, we measured the transmittance of the solution with melting GenNLC-Dex-Mox hydrogel, which reflects the transmittance of aqueous humor after the GenNLC-Dex-Mox multi-drugs delivery system being injected into the desired place. Although the transmittance of released medium decreased down to 62.15 ± 0.12% at 400 m, the transmittance were all above 90% (93.44 ± 0.33% to 100%) at range of 430 nm to 800 nm, which illustrated that the release of hydrogel did not influence clear vision (Fig. 2e).

**Table 3:** The results of viscosity and adhesive abilities of the GenNLC-Dex-Mox hydrogel with or without CaCl_2_.

Samples	Viscosity (Pa.s)	Hardness (g)	Adhesiveness (g)	Stringiness (mm)
Gel with CaCl_2_	6.135	54.00	22.00	3.14
Gel without CaCl_2_	4.336	48.50	20.14	2.26

### In vitro* release studies*

The in vitro drug release profiles of the multi-drug delivery system in vitro are depicted in Fig. 2f. In our drug delivery platform, as shown in the graph, moxifloxacin showed a designed quick release at a constant rate for up to 10 days. Nearly 99% moxifloxacin drug molecules (98.76 ± 2.31%) were released at day 9. About 50% of dexamethasone was released from the thermoresponsive in situ hydrogel at a constant rate in the first week, then, released sustainably with a tapering down rate till day 30 (97.14 ± 3.38%). Moreover, genistein showed the slowest but persistent release profile from the beginning of the assay with a cumulative release of 63.35 ± 2.49% till the final test point (40 days), which means the release of genistein could last for at least two months. Importantly, the releasing profile of genistein was down drifting, which means the release can be long lasting. The results demonstrated that the multi-drug delivery system has a desirable release profiles with programmed release amounts and durations that ideally matched the needs of clinical practice.

### Inhibition effects of GenNLC and GenNLC-Dex-Mox hydrogel on cell viability

**Figure 3:**
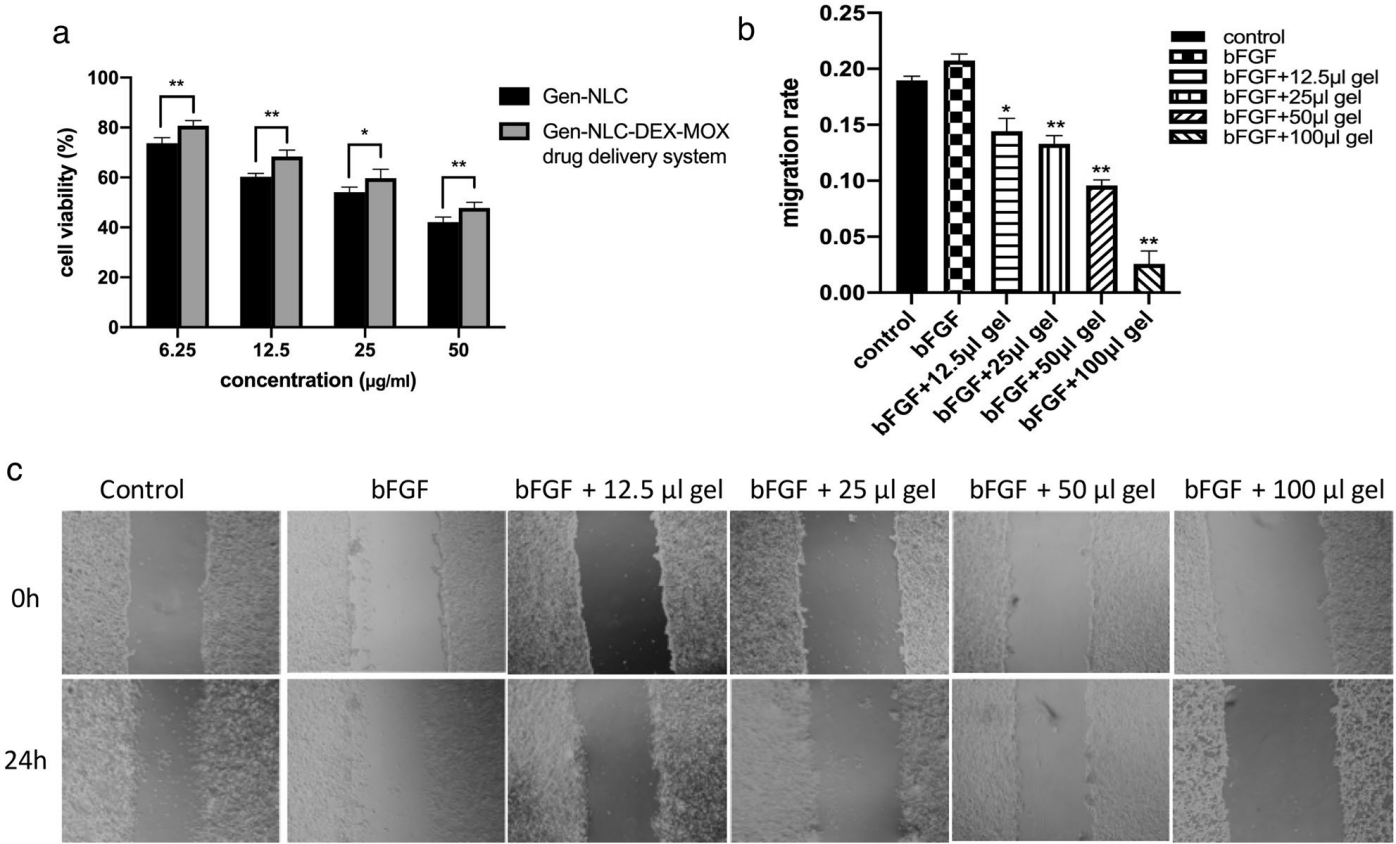
Effects of GenNLCs and GenNLC-Dex-Mox drug delivery system on cell viability and cell migration of LECs. (**a**) GenNLCs and the multi-drug delivery system could inhibit the proliferation of lens epithelial cells in a dose-dependent manner (6.25, 12.5, 25, 50 μg/mL of Genistein concentration in culture medium). However, compared with the results of the groups treated with GenNLCs (73.74**–**42.09%), the cell viabilities in the hydrogel groups were significantly higher (80.75**–**47.78%) (*P* < 0.05). Results are shown as mean ± SD (n = 4). (**b**,**c**) Effects of GenNLC-Dex-Mox treatment on SRA cells migration, scale bar = 100 μm. The wounded area of SRA cells was quantified by ImageJ software. The LECs in the bFGF group had the highest migration rate (20.75%), while, the hydrogel inhibited the cell migration in a dose dependent manner. Results are shown as mean ± SD (n = 3). *P < 0.05, **P < 0.01, ***P < 0.001.

CCK8 assay was used to quantify the inhibition effect of GenNLCs and GenNLC-Dex-Mox thermoresponsive in situ hydrogel on the viability of lens epithelial cells. SRA 01/04 cells were incubated with different concentrations of GenNLC or GenNLC-Dex-Mox gel. The results showed that both GenNLC and our multi-drug delivery system could inhibit the proliferation of lens epithelial cells in a dose-dependent manner (6.25, 12.5, 25, 50 μg/mL of genistein in culture medium). However, compared with those of the groups treated with Gen-NLC alone (73.74 ± 2.19% in the 6.25 μg/mL group, 60.33 ± 1.26% in the 12.5 μg/mL group, 47.78 ± 2.25% in the 25 μg/mL group), the cell viabilities in the hydrogel groups were higher (80.75 ± 2.02% in the 6.25 μg/mL group, 68.36 ± 2.63% in the 12.5 μg/mL group, 54.12 ± 1.97% in the 25 μg/mL group) (*P* < 0.05) (Fig. 3a).

### Inhibition effects of GenNLC-Dex-Mox hydrogel on cell migration

Next, a wound-healing assay was used to detect the inhibitive effect of migration capability of GenNLC-Dex-Mox hydrogel treatment on lens epithelial cells. As depicted in the cell micrographs, the inhibitory effects of the hydrogel on cell invasion and migration were visible. The quantified results showed that the migration rates in all the GenNLC-Dex-Mox in situ gel treatment groups were slowed down in a dose-dependent manner (0.14 ± 0.011 in the 12.5 μL group, 0.13 ± 0.007 in the 25 μL group, 0.096 ± 0.005 in the 50 μL group, 0.026 ± 0.012 in the 100 μL group), compared with that of the bFGF group (0.21 ± 0.006) (*P* < 0*.*05) (Fig. 3b,c). In the group treated with 100 μL hydrogel, it was visible that the density of LECs decreased.

### Inhibition effects of GenNLC-Dex-Mox hydrogel on LECs EMT

**Figure 4:**
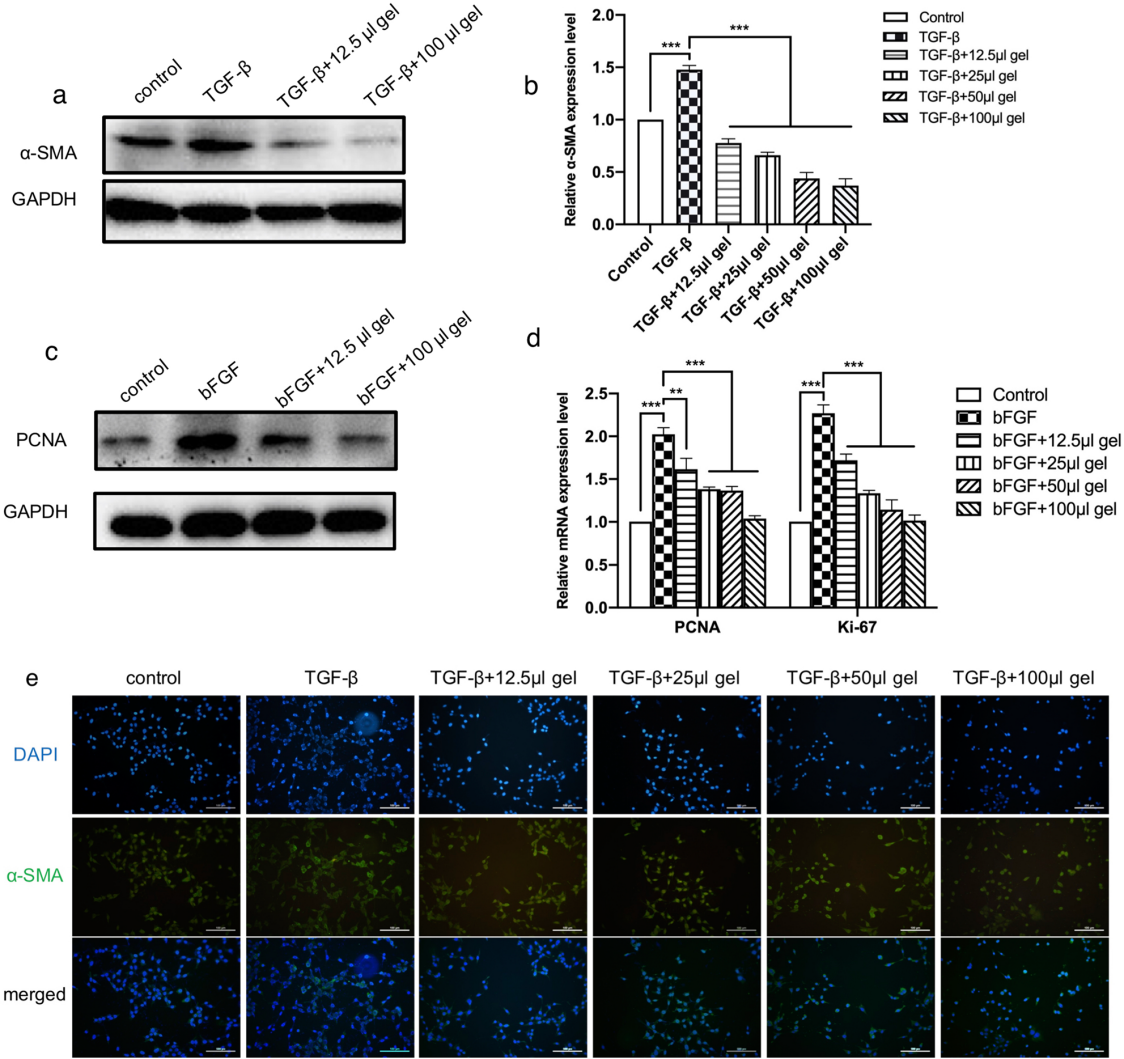
GenNLC-Dex-Mox hydrogel dose dependently inhibited SRA cell proliferation and EMT. The results of western blotting (**a**) and RT-PCR (**b**) both illustrated decreased expression of α-SMA in lens epithelial cells treated by the GenNLC-Dex-Mox thermoresponsive in situ gel in a dose-dependent manner. (**c**,**d**) PCNA and Ki-67 are cell proliferation-associated proteins. The GenNLC-Dex-Mox hydrogel treatment dose-dependently reduced the expression levels of PCNA as well as those of Ki-67. (**e**) Immunofluorescence illustrated the same trends of decreased expression of α-SMA protein in lens epithelial cells treated by increasing doses of the GenNLC-Dex-Mox thermoresponsive in situ gel. Results are shown as mean ± SD (n = 3). *P < 0.05, **P < 0.01, ***P < 0.001.

Furthermore, expression of α-SMA, a symbolic marker of EMT , was detected by western blotting and immunofluorescence. All groups except the blank control were treated by TGF-β, a typical growth factor to induce EMT changes^30,31^. The results of western blotting (Fig. 4a) and immunofluorescence (Fig. 4e) both illustrated a decrease of α-SMA expression in lens epithelial cells treated with the GenNLC-Dex-Mox thermoresponsive in situ gel in a dose dependent manner, opposite to the overexpression of α-SMA in the TGF-β treat alone group. Similar outcomes were noted in the RT-PCR results of α-SMA expression in each experimental group. After being treated with TGF-β, the α-SMA expression level of SRA cells was 1.48 ± 0.04, which was increased comparing with those of the control group (*P* < 0.001). While, compared with the TGF-β alone group, the α-SMA expression levels in the GenNLC-Dex-Mox thermoresponsive in situ gel treatment groups were down-regulated in a dose dependent manner (0.78 ± 0.04 in the 12.5 μL group, 0.66 ± 0.03 in the 25 μL group, 0.44 ± 0.06 in the 50 μL group and 0.37 ± 0.06 in the 100 μL group) (*P* < 0.001) (Fig. 4b).

### Inhibition effects of GenNLC-Dex-Mox hydrogel on cell proliferation

PCNA and Ki-67 are cell proliferation-associated proteins^32–34^. As shown in the western blot graph, the expression of PCNA was upregulated in the bFGF group, and downregulated by treating the lens epithelial cell line with GenNLC-Dex-Mox hydrogel at different concentrations (Fig. 4c). The qRT-PCR results showed the same trends. There was substantial upregulation of PCNA and Ki-67 in the bFGF group (2.03 ± 0.08 and 2.27 ± 0.10 respectively) compared with the control group (*P* < 0.001), which could be reversed by GenNLC-Dex-Mox hydrogel treatment in a dose-dependent manner (PCNA: 1.61 ± 0.13 in the 12.5 μL group, 1.38 ± 0.02 in the 25 μL group, 1.36 ± 0.05 in the 50 μL group, 1.04 ± 0.03 in the 100 μL group; Ki-67: 1.72 ± 0.07 in the 12.5 μL group, 1.33 ± 0.03 of the 25 μL group, 1.14 ± 0.11 in the 50 μL group and 1.02 ± 0.06 in the 100 μL group) (*P* < 0.01) (Fig. 4d).

## Discussion

In this study, we constructed an intracameral temperature-sensitive in situ hydrogel carrying genistein nanostructured lipid carrier, dexamethasone and moxifloxacin with designed release amounts and with lasting duration, which could be injected into the anterior chamber at the conclusion of cataract surgery to reduce inflammation, prevent infection and reduce the incidence of posterior capsular opacification. These results suggest the potential of reducing the need for eye drops and reduce both the short and long term complications following cataract surgery, not only for adults, but also in the pediatric population where post-op drops can be more challenging for compliance. The final concentrations of the three agents in our GenNLC-Dex-Mox temperature sensitive in situ hydrogel with ultimate recipe were 4 mg/mL of dexamethasone, 2 mg/mL of moxifloxacin, and 1 mg/mL of genistein respectively. No burst release was noted for all three imbedded agents. Moxifloxacin showed a designed quick release within 10 days. Half of dexamethasone released from the hydrogel in the first week at a constant velocity, followed by a sustainable release until day 30 with a tapering down manner. Genistein showed a persistent release profile for at least 2 months without burst release. The in vitro results demonstrated the inhibitive effects of the drug delivery system on PCO.

We remodified the genistein nanostructured carrier formulations, which was first designed by our group and had been published previously^23–26^. Compared with the data in our previous studies, this novel GenNLCs had smaller particle sizes in average (39.47 nm *vs.* 80.12 nm), and better stability (stay visibly clear for more than one month *vs.* less than 10 days) without compromise the encapsulation efficiency (92.75% *vs.* 91.14%). Our former formulation of lipids for GenNLCs was Compritol 888 ATO, Gelucire 44/14, and Miglyol 812 N. We modified the recipe by using Compritol 888 ATO and medium-chain triglyceride (MCT) as the lipids in the new formulations based on the results of lipid screening results. These findings suggest the new formulation has substantial improvement in stability. It was suggested that 20–25 mv was an arbitrary value for an incipient stable system that separates low-charged surfaces from highly-charged surfaces, because it was believed that colloids with high zeta potential (negative or positive) are electrically stabilized while colloids with low zeta potentials tend to coagulate or flocculate. The new GenNLCs has a relatively smaller absolute surface charge value compared with the former one (− 4.32 mv *vs*. − 25.08 mv). Although the absolute potential surface value of the new formulation was smaller than that of the previous one, the even higher stability may be explained not only by the combination of Compritol 888 ATO and MCT as the lipids, but also by the introduction of Kolliphor HS 15 as one of the emulsifiers and surfactants, and the introduction of mPEG-PLA for modifying the particle surface. mPEG-PLA has been extensively studied for ocular drug delivery as it is approved by the FDA to make NLCs for clinical administration. mPEG-PLA is an amphiphilic block copolymer with biodegradable, biocompatible and nonantigenic properties, which can provide a sufficiently dense coating to shield the nanoparticle cores from interactions and make smaller-size NLCs with desired drug release kinetics achievable^35,36^. Another major modification in GenNLC preparation was the way to emulsify NLC. Homogenization and ultrasound emulsification are widely used as joints^37^. Because two different kinds of emulsifier, Kolliphor HS 15 and Cremphor EL were used in our study, both of which are powerful emulsifiers^38,39^, we only used hot homogenization method to simplify the procedure of GenNLC preparation without adding ultrasound emulsification method for avoid unstable high surface energy^40^.

Based on the modified GenNLCs, we developed a thermosensitive triple-drug delivery system, GenNLC-Dex-Mox in situ hydrogel, by adjusting the concentrations of F127/F68 as matrix. This in situ hydrogel carried dexamethasone, moxifloxacin and genistein with three different imbedding methods which had not been reported previously after we did a thorough literature research. Genistein was entrapped in NLCs and dexamethasone was combined with CaCl_2_, while, moxifloxacin was added directly into the hydrogel. This special design enabled the multi-drug gel had different programmed release profiles according to the clinical needs. As we mentioned in the introduction part, in clinical practice, antibiotics eye drops are usually applied for a week and topical corticosteroids are used for a month with tapering frequency. For PCO prevention, the longer release duration, the better. The direct encapsulation of moxifloxacin into the thermosensitive hydrogel resulted a shorter release duration for 9 days. Calcium ions was added for coordinating dexamethasone to form supramolecular hydrogel and to control the release of dexamethasone in our strategy^41^. We successfully increased the cross-linking density with better hardness (48.5 g), adhesiveness (20.1 g), and stringiness (2.26 mm) compared with those of the gel without CaCl_2_. These features made it possible to sustainably release dexamethasone in a biphasic drug release pattern which allowed a relatively burst release of 50% dexamethasone in the first week, followed by a slowing down release of the rest of dexamethasone in the next three weeks. Moreover, the benefits achieved by adding CaCl_2_ are not only embodied in the controllable release velocity, but also in the feasibility to use the gel as an injectable system delivered into the anterior chamber by a syringe because of the low viscosity. The release of genistein featured a double-controlled release process in our formulation. The genistein NLCs stayed stably inside the crossed-linking hydrogel, released slowly to the outside medium when the gel was dissolved gradually. Then, genistein went through another round of controlled release from genistein-NLCs into the medium. The double-controlled release enabled the genistein in our multi-drug hydrogel being released at a constantly slow velocity for more than 2 months, which stood a better chance to prevent the formation of posterior capsule opacification caused by the proliferation, migration and EMT transition of the residual lens epithelial cells in the equator of crystal lens. The novel GenNLC-Dex-Mox hydrogel successfully matches the desired administration duration of different eyedrops which are currently used in clinics.

Ideal loading amounts of the working agents in our in situ hydrogel formulation were chosen by calculating the working concentrations and application frequency of dexamethasone and moxifloxacin eyedrops. In each intracameral working dose of the hydrogel (100 μL), 400 μg of dexamethasone, 200 μg of moxifloxacin and 100 μg of genistein were loaded. The loading dose of dexamethasone in our triple drug delivery system is similar to that of DEXYCU, an anterior chamber intracameral dexamethasone drug-delivery suspension, which was approved by FDA as an alternative to corticosteroid drop application in patients undergoing cataract surgery. In phase III clinical trials of DEXYCU, 5 μL injections of 342 or 517 μg DEXYCU dexamethasone drug delivery suspension were compared. Both dosages demonstrated effectiveness in treating inflammation after cataract surgery, while, less adverse events occurred in the 342 μg group^14^. Surodex only loads 60 μg of dexamethasone, which is much less than our formulation, and can only last 10 days in the anterior chamber^42,43^. There is no intraocular antibiotics drug delivery system available in the market. We multiplied the working concentrations of moxifloxacin eyedrops in the anterior chamber with the application frequency and days of instillation, and taking the constant turnover of the anterior aqueous humor into account, 200 μg of moxifloxacin were chosen. When setting the loading amount of genistein, we referred to the in vivo results of the GenNLCs-intraocular lens (IOL) drug delivery system we developed previously (Partial data were not published, under review). According to the amounts of 10 μg genistein loaded into the intraocular lens in our previous drug delivery system which released for more than a week in the anterior chamber, we chose 100 μg genistein for long-lasting release more than 2 months. The in vitro release results of the multi drug delivery system showed that the accumulated releasing amount in aqueous humor was 392.64 μg of dexamethasone at day 9, 197.52 μg of moxifloxacin at day 30 and 63.35 μg of genistein at day 40, which demonstrated excellent performance of the novel triple-drug delivery system.

Other properties which are very important for the intracameral design of the thermoresponsive in situ hydrogel are the gelation temperature and the gelation time. If the gelation time is too high, the drug delivery system may encounter difficulties to form gel inside of the residual lens capsule bag, which means there is possibility for the gel flowing outside of the lens capsule bag, moving into the anterior chamber, resulting increase of intraocular pressure (IOP). On the contrary, if the gelation temperature is too low, it may form non-flowable gel during the process of injecting the in situ gel solution into the lens capsule bag at the end of cataract surgery. By adjusting the percentages of F127 and F68, the final gelation temperature and gelation time of our thermoresponsive in situ hydrogel were above 32 °C, 20 s, respectively. The temperature of ocular surface is 33°C^44^. In addition, there is constant irrigation liquid at room temperature irrigated into the eye during cataract surgery, the actual temperature in the anterior chamber during the surgeries is lower than 36 °C. Meanwhile, because we aimed to make this hydrogel sticking on the posterior capsule, the gelation time should not be too long. Human body temperatures of 35–37 °C or eye surface temperature of 33–34 °C were set as gelation temperatures in some studies^45,46^. With our final formulation, the GenNLC-Dex-Mox thermoresponsive in situ gel presents as liquid at room temperature, while, it forms a non-flowable formulation once being warmed up above 32 °C. The viscosity and adhesiveness results of the gel were suitable for an injectable drug depot. Although the transmittance of GenNLC-Dex-Mox thermoresponsive in situ gel declined in the range of visible light wavelength (400 to 800 nm), the overall transmittance was still over 65% in the range of 500**–**800 nm, which enabled the transmission of part of the visible light for nearly clear vision^47^. However, as we mentioned, the gel was not designed to be applied in the passway of visual axis. It was designed to be injected into the equatorial part of the residual lens capsule bag, which is blocked by the pupil. Thus, we measured the transmittance of the solution with melting GenNLC-Dex-Mox hydrogel, which reflected the transmittance of aqueous humor after the GenNLC-Dex-Mox multi-drugs delivery system being injected into the desired place. The transmittance of released medium were all above 90% in the range of 430 to 800 nm, demonstrating that the release of hydrogel did not influence clear vision.

The in vitro results of the effects of the in situ hydrogel on lens epithelial cells demonstrated that the drug delivery system could effectively inhibit the proliferation, migration and EMT changes of lens epithelial cells in a dose-dependent manner. Although in CCK8 test, compared with the group treated with GenNLCs alone, the cell inhibiting ability in hydrogel group was slightly lower, this might be explained by the double controlled release mode of genistein in GenNLC-Dex-Mox hydrogels, which slowed down the release of genistein into the cell culture medium. The CCK8 only tests the cell viability in 24 h, while, anti-PCO treatment is a long-lasting process. Double controlled release of genistein may yield better long-term results in terms of inhibiting LECs proliferation. The inhibitive effects of the GenNLC-Dex-Mox thermoresponsive in situ gel on PCO were also confirmed by the down-regulated expression of PCNA and Ki-67, which are cell proliferation markers, and the decreasing expression of α-SMA, a marker of LECs EMT. As the anti-infective effects of moxifloxacin and anti-inflammatory effects of dexamethasone have been extensively studied and confirmed, we did not include the results of the anti-infective and anti-inflammatory effects of the novel GenNLC-Dex-Mox thermoresponsive in situ gel.

Over the past few decades, investigations of ocular drug delivery system mainly focused on delivering mono or dual agents. Most of the studied agents are corticosteroids or non-steroidal anti-inflammatory drugs (NSAIDs), and are mainly formulated for ocular surface application^48,49^. Very few studies explored triplet drugs delivery, especially for intracameral injection. Dr. Mohammadi and his colleagues built a thermoresponsive hydrogel that contained levobunolol, dexamethasone and moxifloxacin as an alternative of eyedrops for postoperative treatment in ophthalmic field, which is the only paper we found after doing a thorough review of papers^17^. The depot was an intravitreal design, which is different from our study. Corticosteroid eyes drops were used with a decreasing dose for one month after cataract surgery. Therefore, the chance of steroid-induced ocular hypertension in postoperative period is very unlikely and most of such circumstances do not need to use anti-glaucoma treatment as described in the study by Chen P.Q *et al*^50^.

There are limitations of this study. First, in vivo release profiles are not included in this article, and the in vivo inhibitive effects of the temperature-sensitive in situ hydrogel in animal models of cataract surgery are not included in this study. Further studies to evaluate the in vivo pharmacodynamics, pharmacokinetic effects of the GenNLC-Dex-Mox thermoresponsive in situ hydrogel in animal models are warranted.

In conclusion, we modified a genistein nanostructured lipid carrier, and built an easily injectable temperature-sensitive in situ hydrogel carrying genistein nanostructured lipid carrier, dexamethasone and moxifloxacin, which can be injected into the anterior chamber at the conclusion of cataract surgery especially in the pediatric population, to reduce inflammation, prevent infection and reduce the incidence rate of PCO. We optimized the formulation of the multi-drug delivery system to meet the designed programmed release amounts and times, and evaluated the performance of the drug delivery system in vitro. Such a depot has the potential to replace or cut down the application of eye drops after cataract surgery is performed. Moreover, it has the potential to prevent the formation of posterior capsule opacification.

## Methods

### Materials

Dexamethasone sodium phosphate and moxifloxacin hydrochloride were purchased from Dalian Meilun Biotechnology Co. Ltd., China, and genistein was obtained from Xian Xiaocao Plant Technology Co., Ltd., China. Compritol ATO 888 and Precirol ATO 5 were purchased from Gattefosse, France. Soybean oil was obtained from YiPusheng Pharmaceutical Co. Ltd., China and medium-chain triglyceride (MCT) was procured from CREMER OLEO GmbH & Co. KG. mPEG (2000)**–**PLA (2400) were synthesized by Changchun Institute of Applied Chemistry Chinese Academy of Science. Cremphor EL, Pluronic F68, Pluronic F127 and Kolliphor HS 15 were purchased from BASF corporation, Germany and anhydrous calcium chloride (CaCl_2_) was provided by Tianjin Kermal Chemical Reagent Co. Ltd., China. Phosphate buffer saline (PBS) and Dulbecco’s minimum essential medium (DMEM) were purchased from BOSTER Biological Technology Co. Ltd., China**.** Basic fibroblast growth factor (bFGF) and transforming growth factor-β (TGF-β) were obtained from Gibco, China. Alpha-smooth muscle actin (α-SMA) antibody was provided by Abcam Corporation and proliferating cell nuclear antigen (PCNA) antibody, anti-Ki-67 antibody, GAPDH antibody were purchased from Proteintech Group. Inc. Acrylic Aspheric Intraocular Lens (IOL) were acquired from USIOL, Inc. All other reagents were of analytical grade.

### Preparation of GenNLCs and stability test

GenNLCs were prepared by homogenization emulsification method. We modified the former GenNLC formulations generated by our group, which have been published previously^23–26^, by introducing HS 15 as one of the emulsifiers, adding mPEG-PLA and screening lipids. Compritol ATO 888 and Precirol ATO5 were chosen to be tested as the solid lipids, while MCT and soybean oil were selected to test as the liquid lipids, as these lipids are widely studied in the formulation of nanostructured lipid carrier drug delivery platform. The quantities and the types of other ingredients were fixed while we kept the proportion of the lipids but changed their matching. Briefly, 225 mg Crempher EL was homogenized with 5 mL ultrapure water at 87 °C for 10 min by using magnetic stirrer. Meanwhile, 10 mg genistein, 75 mg HS 15, 50 mg mPEG-PLA and lipids (shown in Table 1) were dissolved by magnetic rotor slowly at 87 °C as well and the hot Crempher EL premix was added immediately into it. After homogenized at 87 °C for 7 min, the GenNLCs were cooled in ice water and stored at 4 °C. The stability of GenNLCs was measured by testing how long the NLCs stored at 4 °C could stay clarification without sediments.

### Particle size and zeta potential measurement

Malvern Zetasizer Nano ZS instrument was used for measuring the average particle size, polydispersity index (PDI), and zeta potential of the GenNLCs. The measurements were done at room temperature^26^.

### Morphology observations of GenNLCs

Transmission electron microscopy (TEM) (JEM-2100,7 Japan) was used for observing the morphology of GenNLCs. Sample was placed on a copper grid for adhering for 30 s. Removing excess dispersion with filter paper was necessary before and after adding 2% phosphotungstic acid solution for staining^26^.

### Evaluation of encapsulation efficiency of genistein-NLC

Quantification of encapsulation efficiency (EE%) of genistein in GenNLCs were determined by high performance liquid chromatography (HPLC) method^26,51^. Sephadex G-50 was added into a 2.5 mL syringe and centrifugated at 2000 rpm for 2 min. Then, 0.2 mL of GenNLCs was added to the column and centrifugated at 2000 rpm for 2 min and washed by distilled water three times. Next, the eluent was collected and destroyed by using dichloromethane and methanol mixture (1:4, v/v). The mobile phase was made up of methanol-0.05% phosphoric acid aqueous solution (70/30, v/v) with a flow rate of 1.0 mL/min. The quantity of encapsulated genistein was measured by HPLC with Kromasil 100-5C18 column (250 mm × 4.6 mm) at 270 nm, 37 °C. Another 0.2 mL of GenNLCs was added to the organic reagent to account for any destroyed in the previous process. The EE of NLCs was calculated using the following Eq. (1):

1 $${\text{EE }}\left( \% \right) = \frac{encapsuled\; drug}{{{\text{total}}\;{\text{amount}}\;{\text{of }}\;{\text{drug}}\;{\text{added}}\;{\text{at}}\;{\text{beginning}}}}\underline { \times } 100\%$$

### Formation of GenNLC-Dex-Mox hydrogel

Different weight/volume percentage concentrations of F127 and F68 combinations in demand were dissolved by 4 mL ultrapure water (half of the final hydrogel volume) and stored at 4 °C overnight. On the second day, 32 mg of dexamethasone sodium phosphate, 16 mg of moxifloxacin hydrochloride and 12.8 mg of CaCl_2_ were mixed into 4 mL GenNLC. The mixed solutions were added dropwise into the prepared F127/F68 gel solution and then the mixture was dispersed by magnetics stirring in ice water for 2 h. The final concentrations of these three components in our GenNLC-Dex-Mox multi-drug delivery system were 4 mg/mL of dexamethasone, 2 mg/mL of moxifloxacin, and 1 mg/mL of genistein respectively.

### Gelation temperature and gelation time

Gelation temperature and gelation time were detected by using the qualitative vial inversion test. One milliliter GenNLC-Dex-Mox delivery system was added into a glass tube which was bathed in the water with low temperature. The tube was heated gradually at an increase of 1 °C each time, followed by continuously tilting the tube approximately 90° during the test. When the solution was not able to flow, the drug delivery system was considered to transform to gel. The temperature at which the sample transformed to gel was recorded as gelation temperature. Similarly, the tube was incubated in 32 °C water and the duration for the liquid transforming to immovable gel phase was recorded as gelation time. Each sample was measured at least three times.

### Viscosity and adhesive properties

Quantitative rheological measurements were performed to test viscosity by using rheometer (AR2000ex, TA company, USA). Briefly, 5 mL hydrogel was dropped in the middle of the plate, pressed and tested by using a flat head with a diameter of 40 mm. At 25 °C, the experiment was done with a shear rate of 0–120 s^−1^. Adhesive ability of GenNLC-Dex-Mox hydrogel were detected by TA. XT. Plus Texture Analyser with a flat-cap. Experimental parameter were set as follows: pretest speed = 1 mm/s, test speed = 1 mm/s, back speed = 1 mm/s. Tests were conducted at 37 °C. Adhesiveness properties were reported as hardness, adhesiveness and stringiness.

### Transmittance

The UV–vis spectrophotometer (UNIC UV2800A, China) was used to measure the transmittance of GenNLC-Dex-Mox hydrogel. The samples were injected into a quartz cuvette and the values were measured by a spectrophotometer at wavelengths ranging from 400 to 800 nm.

### In vitro* drug release studies*

To mimic the release in vivo, 200 μL GenNLC-Dex-Mox hydrogel were placed into a 1.5 mL centrifuge tube and incubated at 37 °C for 30 min. Next, 1.2 mL PBS was poured into the tube as the release medium then put the tubes back into the air shake bath for an incubation temperature of 37 °C. At each time point, 1 mL of media was replaced by fresh PBS for further drug release^17^. The collected medium containing the released drugs was determined by the HPLC method separately. The collected medium containing the released drugs was determined by the HPLC method separately. The genistein was detected with the same conditions mentioned in EE% measurement part. The Kromasil 100-5C18 column (250 mm × 4.6 mm) was also used for dexamethasone and moxifloxacin. For dexamethasone, the mobile phase was composed of methanol and monopotassium phosphate buffer (0.05 mol/L) in the ratio of 70:30% v/v. The column was maintained at 30 °C and the emission wavelength was at 240 nm. For moxifloxacin, the mobile phase was a 34:66 (v/v) mixture of methanol and 0.025 mol/L monopotassium phosphate buffer (containing 0.2% triethylamine). The temperature of the column for moxifloxacin was set as 40 °C and the emission wavelength was at 290 nm. The mobile phase pumped at a flow rate of 1.0 mL/min.

### *Cell culture and CCK*8* assay*

A lens epithelial cell line, SRA 01/04 cell line, was cultured in DMEM supplemented with 10% FBS, penicillin and streptomycin in a humidified atmosphere of 5% CO_2_ at 37 °C. SRA cells were seeded at a density of 2.5 × 10^4^ cells/well in 96-well plates with 100 μL culture medium and incubated 4 h for cell attachment. After cells were adherent, medium was replaced with serum-free DMEM for 12-h starving culture. Cells were treated with different concentrations of GenNLCs (0, 6.25, 12.5, 25 and 50 μg/mL of genistein), or different doses of GenNLC-Dex-Mox multi-drug delivery system (0, 0.625, 1.25, 2.5, and 5 μL) for 24 h. Next, 10 μL of CCK8 solution was added to each well that contained 100 μL fresh DMEM, and the plate was incubated for 2 h at 37 °C. Cell viability was determined by scanning with a microplate reader at 450 nm. The experiment was repeated at least three times.

### Wound healing test

SRA cells were seeded into 6-well plates at a density of 5 × 10^5^ cells/well. Three hours after attachment, cells were cultured in starving culture medium for another 12 h as mentioned above. A pipette was used to scratch across the surface to make a wound. In the control group, 10 ng/μL bFGF was added to the culture medium without other treatment. In the experimental groups, 10 ng/μl bFGF plus different dosages of GenNLC-Dex-Mox multi-drug delivery system (12.5, 25, 50 and 100 μL) were added into the culture medium. Cells were incubated for another 24 h, then, the areas of the scratching wounds were measured and analyzed by Image J software (version: v1.52q, Wayne Rasband National Institutes of Health, USA)^52,53^.

### Epithelial mesenchymal transition (EMT)

SRA 01/04 cells were seeded into 6-well plates at a density of 5 × 10^5^ cells/well. Three hours after cells were attached, culture medium was switched to serum free medium and cells were cultured for another 12 h. Next, 10 ng/μL TGF-β was added to the culture medium in the control group. In the experimental groups, 10 ng/μL TGF-β plus different dosages of GenNLC-Dex-Mox multi-drug delivery system (12.5, 25, 50 and 100 μL) were added into the culture medium. Cells were continuously incubated for another 24 h and harvested for western blot, immunofluorescence staining and quantitative polymerase chain reaction (qPCR).

### Proliferation test

SRA 01/04 cells were prepared in the same way as mentioned above. Then, 10 ng/μL bFGF was added to the culture medium in the control group. In the experimental groups, 10 ng/μL bFGF plus different dosages of GenNLC-Dex-Mox multi-drug delivery system (12.5, 25, 50 and 100 μL) were added into the culture medium. Cells were continuously incubated for another 24 h and harvested for western blot and RT-qPCR.

### Western blot and RT-qPCR

Proteins were extracted and separated by 12% SDS PAGE, then, was blotted onto PVDF membranes. The blots were cut according to the weight of target proteins prior to hybridization with antibodies. After being blocked by 5% non-fat milk, PVDF membranes were treated with specific primary antibodies overnight at 4 °C. Secondary antibodies were subsequently used for incubation and an ECL kit was applied to visualize the signal blots. Total RNA was isolated by Trizol from cells. cDNA was synthetized and SYBR GREEN was applied for qPCR with cDNA as templates and GAPDH as internal control. Primer sequences were designed as following:

- α-SMA, F: CCCAGCCAAGCACTGTCA, R: TCCAGAGCAGCACGATG;
- PCNA, F: GCGCTAGTATTTGAAGCACCAA, R: CGATCTTGGGAGCCAAGTAGTA;
- Ki-67, F: CGATCTTGGGAGCCAAGTAGTA, R: TTGCTGTTCTGCCTCAGTCTT;
- β-actin, F: CATCCGTAAAGACCTCTATGCCAAC, R: ATGGAGCCACCGATCCACA.

### Immunofluorescence

Cells were washed with PBS three times, fixed in 4% paraformaldehyde for 20 min at room temperature and treated with 0.2% Triton X-100 for 15 min on ice. Cells were further blocked with 5% bovine serum albumin (BSA) in PBS for 1 h at room temperature. For immunofluorescence, the cell samples were incubated with the primary antibody overnight at 4 °C and then incubated with secondary antibody for 1 h at room temperature. Nuclear staining was DAPI blue. Images were acquired using Inverted Fluorescence Microscope.

### Statistical analysis

All data were expressed as mean ± standard deviation (SD). Statistical analysis was performed with Student’s *t* test by Graphpad Prism 8.0 software (La Jolla, CA, USA). All *P* values were two-sided; there was no correction for multiple analyses so that no specific *P* value cutoff was used to denote statistical significance.

## Supplementary Information

Supplementary Information.
