# Supplementary material for: Thermoresponsive GenisteinNLC-dexamethasone-moxifloxacin multi drug delivery system in lens capsule bag to prevent complications after cataract surgery
Source: Sci Rep. 2021 Jan 8;11:181. doi: 10.1038/s41598-020-80476-x (PMC7794611; doi:10.1038/s41598-020-80476-x)
Supplement: Supplementary file 1 — Supplementary Information. [file 41598_2020_80476_MOESM1_ESM.pdf]

Thermoresponsive GenisteinNLC-Dexamethasone-Moxifloxacin Multi Drug Delivery System in Lens Capsule Bag to Prevent Complications after Cataract Surgery

Tingyu Yan[1], yantingyu@163.com; Zhongxu Ma[2], mazhongxu@hotmail.com; Jingjing Liu[1], 653673751@qq.com; Na Yin[3], yinna_pharm@163.com; Shizhen Lei[1], lzssyb@163.com; Xinxin Zhang[1], 394338224@qq.com; Xuedong Li[1], sklddfe@126.com; Yu Zhang[3*], pharmzy@163.com; Jun Kong[1*], kongjun@hotmail.com

[1]Department of Ophthalmology, the Fourth Affiliated Hospital of China Medical University. No.11 Xinhua Road, Heping District, Shenyang, Liaoning Province, China 110005;
[2] Tianjin Eye Hospital, Tianjin Key Laboratory of Ophthalmology and Vision Science, Clinical College of Ophthalmology, Tianjin Medical University. No. 4 Gansu Rd, Heping District, Tianjin, China 300020;
[3] Department of Pharmaceutics, Shenyang Pharmaceutical University. No.103 Wen Hua Road, Shenyang, China 110016

Tingyu Yan and Zhongxu Ma contributed equally.

Supplementary information

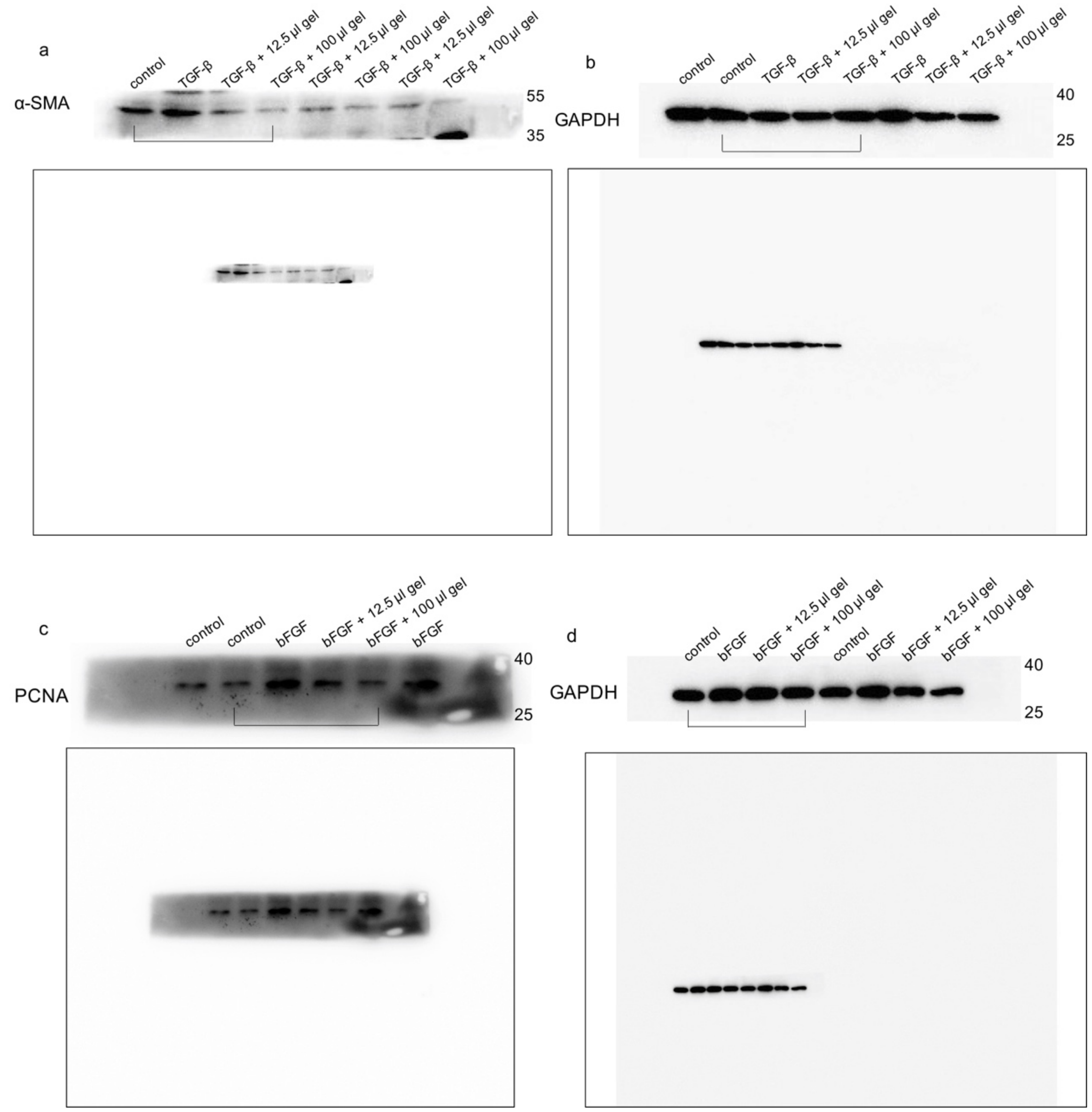

Supplementary Figure 1: Original western blot membranes for membrane strips presented in Figure 4 of the manuscript.
